# Effects of Intensive Insulin Therapy Within One Week on Glycemic Control and β-Cell Function in Patients with T2DM

**DOI:** 10.3390/jcm15218297

**Published:** 2026-10-25

**Authors:** Wei Jiang, Xubin Yang, Beisi Lin, Zhigu Liu, Xuanrong Chen, Shuo Lin, Daizhi Yang, Yintong Huang, Ruofan Wang, Wen Xu, Longyi Zeng

**Affiliations:** 1 Department of General Medicine, The Third Affiliated Hospital of Sun Yat-Sen University, Guangzhou 510530, China; jiangw39@mail.sysu.edu.cn (W.J.); chenxr5@mail.sysu.edu.cn (X.C.); 2 Department of Endocrinology and Metabolism, The Third Affiliated Hospital of Sun Yat-Sen University, Key Laboratory of Diabetology of Guangdong Province, Guangzhou 510630, China; yxbin@mail.sysu.edu.cn (X.Y.); linbs3@mail.sysu.edu.cn (B.L.); linshuo3@mail.sysu.edu.cn (S.L.); yangdzh3@mail.sysu.edu.cn (D.Y.); huangyt275@mail2.sysu.edu.cn (Y.H.); wangrf33@mail2.sysu.edu.cn (R.W.); 3 Department of Endocrinology, The People’s Hospital of Baoan Shenzhen, The Second Affiliated Hospital of Shenzhen University, Shenzhen Hospital of Guangdong Provincial People’s Hospital, The Affiliated Baoan Hospital of Southern Medical University, Shenzhen Baoan Clinical Medical School of Guangdong Medical University, The 8th People’s Hospital of Shenzhen, Shenzhen 518000, China; 13760916206@163.com

**Keywords:** type 2 diabetes, intensive insulin therapy, β-cell function, time in range, glycemic variability

## Abstract

**Objective**: To evaluate the impact of intensive insulin therapy (IIT) within 1 week on glycemic control and β-cell function indices in patients with type 2 diabetes mellitus (T2DM). **Methods**: This retrospective study included 726 patients with T2DM who underwent continuous subcutaneous insulin infusion within 1 week. Patients were divided into three groups based on disease duration: short (≤1 year), medium (>1 and <10 years), and long (≥10 years) duration groups. The primary outcome was the changes in glycemic control and β-cell function indices after IIT. Factors influencing the improvement in β-cell function metrics were also analyzed. **Results**: After IIT within 1 week, fasting plasma glucose (FPG) and postprandial plasma glucose (PPG) levels significantly decreased (*p* < 0.01 for both). HOMA-β, 30 min insulinogenic index, 30 min disposition index, and insulin secretion sensitivity index-2 markedly improved from baseline [37.69 (41.03) VS. 32.40 (46.06), 3.16 (3.97) VS. 2.12 (3.51), 336.85 (397.05) VS. 188.93 (263.45), and 185.92 (122.89) VS. 120.81 (90.12), respectively] (*p* < 0.01 for all). Insulin sensitivity, measured by HOMA-IR and Matsuda index, significantly improved (*p* < 0.01 for both). Significantly greater improvements in glycemic control and β-cell function measures were observed in patients with a shorter diabetes duration. Age, duration of diabetes, BMI, HbA1c, duration of IIT, and time in range were significantly associated with improvements in β-cell function indices. **Conclusions**: IIT within 1 week was associated with improved glycemic control and β-cell function indices in T2DM patients, with the most pronounced benefits observed in those with a shorter disease duration.

## 1. Introduction

Although the comprehensive pathological mechanisms of type 2 diabetes mellitus (T2DM) have not been fully elucidated, the progressive decline in β-cell function is recognized as the key pathological feature of the disease. Moreover, evidence has shown that decreased β-cell function is closely associated with diabetes-related complications [1,2]. Hence, preserving β-cell function is crucial for maintaining long-term glycemic homeostasis and reducing the risk of such complications.

Data from the United Kingdom Prospective Diabetes Study (UKPDS) suggest that β-cell function decreased by 40–50% at the time of T2DM diagnosis and progressively declined over time [3]. Similarly, a cross-sectional study from China showed that β-cell function declined by 2% annually in Chinese patients with T2DM [4]. In the past, β-cell dysfunction was considered irreversible as diabetes progressed. However, numerous studies in recent years have demonstrated that early and aggressive interventions, including intensive insulin therapy (IIT) and metabolic surgeries, can lead to varying degrees of β-cell function improvement [5,6,7]. A previous study proved that 2 to 5 weeks of IIT has favorable outcomes on the recovery and maintenance of β-cell function, as well as prolonged glycemic remission (achieving glycemic control without medications), compared with oral hypoglycemic agents in some patients with newly diagnosed T2DM. Moreover, the relief of glucose toxicity and improvement in β-cell function have also been observed in patients with established T2DM. It was reported that first-phase insulin secretion was partially restored in T2DM patients with a median disease duration of 3 years after an average of 12 days of continuous subcutaneous insulin infusion (CSII) [8]. Attenuation of endoplasmic reticulum stress, reduction in inflammation, promotion of β-cell proliferation, and induction of dedifferentiated β-cell redifferentiation are believed to be responsible for the β-cell function improvement induced by IIT [9,10,11,12].

To date, there is no consensus on the optimal duration of IIT required to improve β-cell function in patients with T2DM. In previous clinical studies, the duration of IIT employed in patients with newly diagnosed or established T2DM ranged from 2 weeks to 3 months. However, whether IIT within 1 week is sufficient to relieve glucotoxicity and improve β-cell-function-related indices has not been studied. Hence, this study aimed to evaluate the effect of IIT within 1 week on β-cell function indices in patients with varying durations of T2DM, and to analyze the factors affecting improvement in β-cell function indices.

## 2. Materials and Methods

### 2.1. Study Design and Population

In this retrospective study, 726 subjects with T2DM who were hospitalized in the Department of Endocrinology and Metabolism at our institution between 1 May 2022 and 24 May 2025 were included. The inclusion criteria were as follows: (1) diagnosed with T2DM according to 1999 World Health Organization criteria; (2) had sufficiently complete electronic medical records during the hospital stay; (3) received CSII using an insulin pump (Paradigm^®^ 722, Medtronic, Northridge, CA, USA) with insulin aspart (Novo Nordisk, Bagsvaerd, Denmark) or insulin lispro (Humalog, Eli Lilly, Indianapolis, IN, USA) within 1 week; and (4) underwent comprehensive evaluation of β-cell function using a mixed meal tolerance test (MMTT) with venous blood samples collected at fasting, 30 min, and 120 min after ingestion to determine plasma glucose, insulin, and C-peptide levels before and after IIT. The exclusion criteria included the following: (1) type 1 diabetes, secondary diabetes, or the presence of positive glutamic acid decarboxylase antibody; (2) severe acute diabetic complications (including diabetic ketoacidosis and hyperosmolar hyperglycemic states); (3) severe infectious diseases; (4) use of drugs that affect glucose metabolism, such as glucocorticoids, etc.; (5) pregnancy or breastfeeding; and (6) severe hepatic, renal, or heart disease.

After finishing routine blood tests, the patients received CSII treatment on the second day of hospitalization. The initial total insulin dose was 0.4–0.6 U/kg/day, with 40% administered as basal insulin (divided into six time periods throughout the day: 00:00 to 03:00, 03:00 to 07:00, 07:00 to 12:00, 12:00 to 17:00, 17:00 to 22:00, and 22:00 to 24:00), and 60% as bolus injections, based on previous research [13]. Patients maintained their prior oral hypoglycemic drugs (OADs) except secretagogues during the IIT period. A baseline MMTT (provided approximately 350 kcal) was performed on the second day after admission before IIT, followed by a post-IIT MMTT the morning after CSII discontinuation and overnight fasting. The interval between MMTTs varied according to IIT duration. During hospitalization, all patients were provided with diets of 25–30 kcal/kg of ideal body weight, consisting of 50–60% carbohydrates, 15–20% protein, and 20–25% fat per day, as recommended by the current Chinese guideline [14]. All meals were consumed within 20 min. No additional food or drink was allowed unless hypoglycemia occurred.

All patients received seven-point capillary blood glucose monitoring (before and 2 h after each meal, and at bedtime) using the blood glucose monitoring device Contour^®^ Plus (Ascensia Diabetes Care, Basel, Switzerland). Insulin doses were adjusted according to seven-point capillary blood glucose profiles to achieve glucose targets, defined as fasting capillary glucose of 4.4–7.0 mmol/L and 2 h postprandial capillary glucose of 4.4–10.0 mmol/L. Hypoglycemia was defined as a capillary blood glucose level of less than 3.9 mmol/L, with or without symptoms. Among these patients, those who underwent continuous glucose monitoring (CGM) with the CGM MiniMed 722 System (Medtronic, Northridge, CA, USA) during CSII were included as a subgroup to analyze the relationship between β-cell function and glycemic variability parameters derived from the CGM system.

### 2.2. Data Collection

Patients’ baseline characteristics, including age, gender, duration of diabetes, and information on prior OADs before admission, were collected. After an overnight fast of 10 h, anthropometric data were measured, and baseline blood samples were taken to measure glycosylated hemoglobin A1c (HbA1c) and lipid profiles. Plasma glucose, insulin, and C-peptide levels at fasting, 0.5 h, and 2 h during MMTT were measured at baseline and after CSII treatment to evaluate β-cell function. To minimize interference from exogenous insulin in the evaluation of β-cell function, CSII was discontinued at 10:00 pm the night before the evaluation of β-cell function.

### 2.3. Evaluation of β-Cell Function and Insulin Sensitivity

Comprehensive indices of β-cell function, including homeostasis model assessment of β-cell function (HOMA-β) [15], 30 min insulinogenic index (IGI_30_) [16], 30 min disposition index (DI_30_) [17], insulin secretion sensitivity index-2 (ISSI-2) [18], Δ Ins_0–120_/Δ Gluc_0–120_ × Matsuda index (MI), and AUC_INS_/AUC_GLU_ were adopted in this study. Insulin sensitivity was evaluated by MI [19] and homeostatic model assessment of insulin resistance (HOMA-IR) [15]. The above indices and their detailed calculations are listed in Supplementary Table S1.

### 2.4. Evaluation of Glycemic Variability

Estimated glycemic variability parameters were derived from the self-monitoring of blood glucose (SMBG) profiles. For each patient, glycemic variability parameters were calculated based on the seven-point capillary blood glucose monitoring profile. These parameters included estimated time above range (eTAR_SMBG_), estimated time in range (eTIR_SMBG_), estimated time below range (eTBR_SMBG_), estimated standard deviation of blood glucose (eSDBG_SMBG_), estimated largest amplitude of glycemic excursion (eLAGE_SMBG_), estimated mean amplitude of glycemic excursion (eMAGE_SMBG_), estimated postprandial glucose excursion (ePPGE_SMBG_), estimated glucose coefficient of variation (eCV_SMBG_), and estimated mean blood glucose (eMBG_SMBG_). eTIR_SMBG_, eTAR_SMBG_, and eTBR_SMBG_ were calculated as the percentages of SMBG measurements with glucose levels within, above, and below the target glucose range of 3.9–10.0 mmol/L, respectively. Specifically, eTIR_SMBG_ was calculated as the number of measurements with glucose levels of 3.9–10.0 mmol/L divided by the total number of SMBG measurements and multiplied by 100%. eTAR_SMBG_ and eTBRS_MBG_ were calculated using the same denominator and multiplier, with glucose thresholds of >10.0 mmol/L and <3.9 mmol/L, respectively. These SMBG-derived indices reflect the proportions of sampled glucose measurements within the respective ranges, rather than the actual percentages of time spent in these ranges as measured by CGM. eMBG_SMBG_ was calculated as the arithmetic mean of all blood glucose measurements; eSDBG_SMBG_ was calculated as the standard deviation of all blood glucose measurements; ePPGE_SMBG_ was calculated as the mean absolute difference between the 2 h postprandial and the corresponding preprandial glucose level for the three main meals; eLAGE_SMBG_ was calculated as the difference between the maximum and minimum blood glucose values; eCV_SMBG_ was calculated as eSDBG_SMBG_ divided by the corresponding eMBG_SMBG_, multiplied by 100%. eMAGE_SMBG_ was calculated as follows: (1) eSDBG_SMBG_ was calculated from all blood glucose measurements; (2) glucose excursions with amplitudes of ≤1 eSDBG_SMBG_ were excluded; and (3) the direction of the first qualifying glucose excursion was used to determine the direction of subsequent excursions. eMAGE_SMBG_ was calculated as the mean amplitude of all qualifying excursions, defined as the absolute difference between peak and nadir glucose levels, with amplitudes of >1 eSDBG_SMBG_ [20]. Although mean eMAGE_SMBG_ can be calculated from SMBG data, intermittent measurements may not capture the full magnitude or frequency of glycemic excursions. Therefore, CGM-derived MAGE may provide a more comprehensive assessment of glycemic excursions. In the subgroup of patients who underwent CGM, glycemic variability parameters were obtained from the CGM system [21]; CGM-derived metrics included TAR_CGM_, TIR_CGM_, TBR_CGM_, SDBG_CGM_, MAGE_CGM_, CV_CGM_, and MBG_CGM_. TIR_CGM_, TBR_CGM_, and TAR_CGM_ were defined as the percentages of time spent within the glucose range of 3.9–10.0 mmol/L, below 3.9 mmol/L, and above 10.0 mmol/L, respectively. CGM-derived metrics were distinguished from the SMBG-derived estimates because they were based on continuous glucose measurements rather than intermittent seven-point SMBG profiles.

### 2.5. Follow-Up

After discharge, patients were instructed to continue dietary and physical activity interventions and to use individualized glucose-lowering regimens based on their glycemic levels, β-cell function, diabetic complications, and comorbidities. Follow-up assessments of FPG and HbA1c were conducted at 3–6 months.

### 2.6. Statistical Analysis

All statistical analyses were performed with SPSS software version 25 (IBM Corporation, New York, NY, USA). Variables were tested for normality. Normally distributed continuous variables were presented as mean ± standard deviation, and non-normally distributed continuous data were presented as median (interquartile range). The paired nonparametric Wilcoxon test (for non-normally distributed variables) or paired Student’s *t*-test (for normally distributed variables) was used to compare changes in β-cell function and insulin sensitivity indicators and glycemic levels after CSII. Normally distributed continuous variables were compared between two independent groups using the independent-samples *t*-test. For comparisons among three independent groups, normally distributed continuous variables were analyzed using one-way analysis of variance (ANOVA), followed by Fisher’s least significant difference (LSD) test for pairwise comparisons, whereas non-normally distributed continuous variables were analyzed using the Kruskal–Wallis test, followed by Dunn’s test for pairwise comparisons. Categorical variables were reported as frequencies and percentages and analyzed with the chi-square test. Adjusted linear regression analyses were performed to compare glycemic variability parameters and post-treatment glycemic levels, β-cell function-related indices, and insulin-sensitivity-related indices among groups. The models were adjusted for clinically relevant covariates, including age, BMI, HbA1c, and glucose-lowering medications administered during IIT. Differences in baseline characteristics between groups were also considered when determining the covariates included in the adjusted models. Multivariable linear regression analyses or binary logistic regression analyses were performed to explore factors associated with absolute and percentage changes in β-cell function-related indices (ISSI-2) from baseline, as well as the likelihood of a ≥2-fold increase in ISSI-2. Age, diabetes duration, BMI, HbA1c, IIT duration, TIR, and MAGE were included as independent variables based on their clinical relevance, evidence from previous studies, and potential confounding effects. TIR and MAGE were derived from SMBG in the entire study population and from CGM data in the CGM subgroup. *p* < 0.05 was considered statistically significant. Missing data were minimal (BMI: 1/726 [0.1%]; HbA1c: 3/726 [0.4%]). Therefore, complete case analyses were conducted without imputation.

## 3. Results

### 3.1. Baseline Characteristics of the Patients

**Figure 1:**
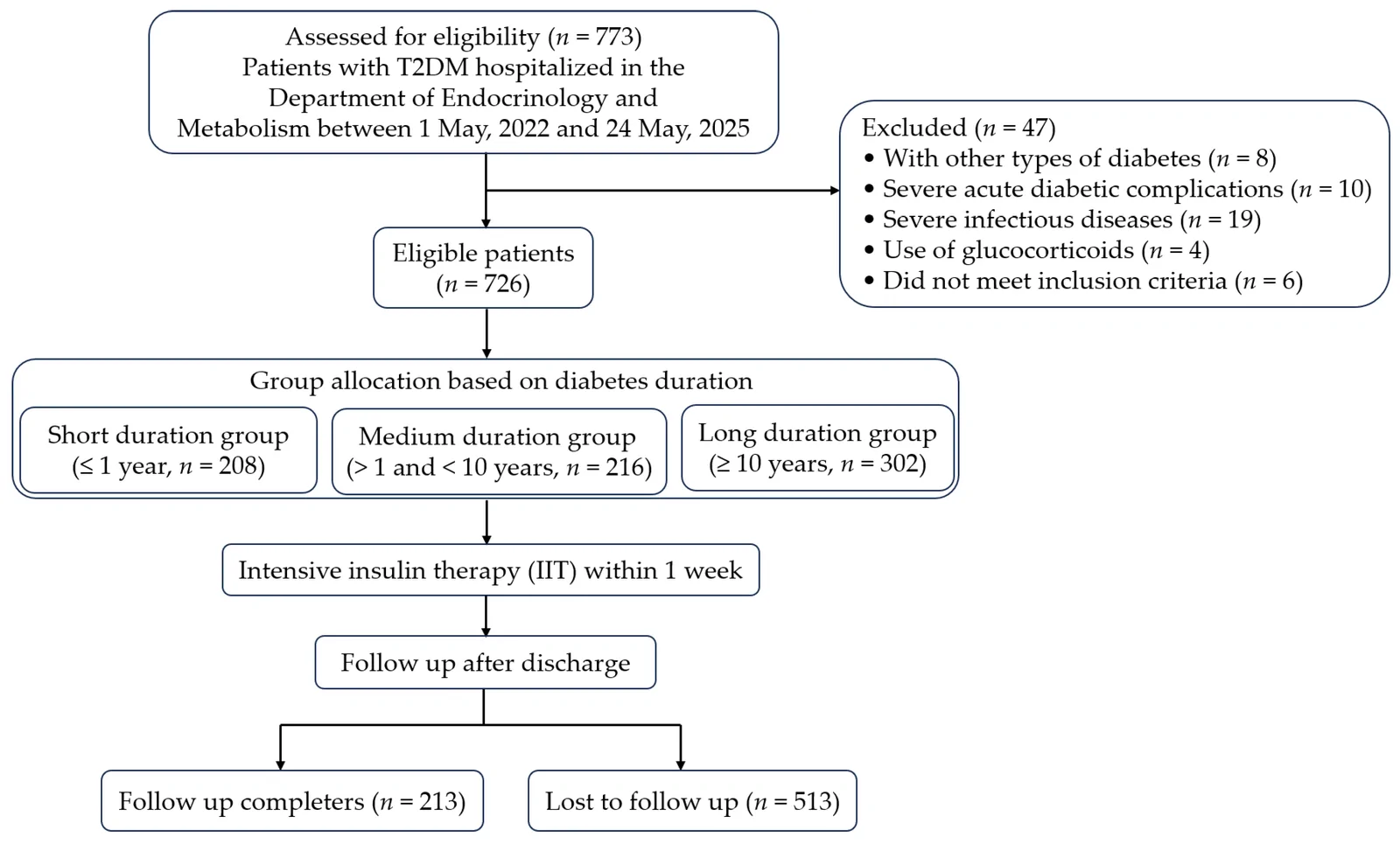
Flowchart of patient inclusion and follow-up.

A total of 726 patients were included in this study. The flowchart of patient inclusion is shown in Figure 1. Patients previously treated with OADs maintained their OAD regimen during the IIT period, except that sulfonylureas and glinides were discontinued, and GLP-1 receptor agonists were withheld. All patients were stratified into three groups based on the duration of T2DM: short duration group (≤1 year), medium duration group (>1 year and <10 years), and long duration group (≥10 years). Age, body mass index (BMI), HbA1c levels, and use of diabetes medications during IIT varied among the three groups (Table 1). All patients received IIT within 1 week. Of these, 0.28% received IIT for 2 days, 4.27% for 3 days, 21.07% for 4 days, 41.32% for 5 days, 22.59% for 6 days, and 10.47% for 7 days. Additionally, fasting plasma glucose (FPG) and 0.5 h postprandial plasma glucose (PPG) levels were different at baseline across groups, with the lowest values observed in the long duration group (Table 2).

### 3.2. Change in Glucose Levels After IIT Within 1 Week

After IIT within 1 week, both FPG and PPG levels significantly reduced in the entire study population and across all diabetes duration subgroups. After adjusting for age, BMI, HbA1c, use of diabetes medications during IIT, and baseline glycemic variables among the three groups, it was found that IIT within 1 week was associated with greater improvements in FPG, 0.5 h PPG, and 2 h PPG in the short and medium duration groups than in the long duration group (Table 2).

### 3.3. Improvement in β-Cell Function and Insulin Resistance After IIT Within 1 Week

Comprehensive indices were utilized to evaluate β-cell function variables in this study. Our findings demonstrated significant improvement in β-cell function indices in all patients, as represented by the increase in multiple β-cell function indices, including HOMA-β, IGI_30_, DI_30_, ISSI-2, Δ Ins_0–120_/Δ Gluc_0–120_ × MI, and AUC_Ins_/AUC_Gluc_ after IIT (Table 3). As shown in Table 3, baseline values of HOMA-β, IGI_30_, DI_30_, and Δ Ins_0–120_/Δ Gluc_0–120_ × MI varied among these three groups with different durations of diabetes. After adjusting for age, BMI, HbA1c, use of diabetes medications during IIT, and baseline β-cell function variables among the three groups, HOMA-β, IGI_30_, DI_30_, ISSI-2, and AUC_Ins_/AUC_Gluc_ in the short duration group, as well as HOMA-β, DI_30_, and ISSI-2 in the medium duration group, were significantly higher than those observed in the long duration group after IIT. Furthermore, HOMA-β, IGI_30_, ISSI-2, and AUC_Ins_/AUC_Gluc_ were significantly greater in the short duration group than those observed in the medium duration group after IIT. Additionally, substantial improvements in insulin resistance metrics after IIT were observed in all patients, as demonstrated by changes in HOMA-IR and MI (Table 3).

### 3.4. Glycemic Variability Among the Three Groups with Different Diabetes Duration

As shown in Table 4, analysis of glycemic variability indices calculated from seven-point capillary blood glucose profiles revealed that the short duration group had significantly higher eTIR_SMBG_ and lower eTAR_SMBG_ and eMBG_SMBG_ levels compared with the medium and long duration groups after adjustment for age, BMI, HbA1c, and the use of diabetes medications during IIT.

Of the 726 patients, 442 patients underwent CGM during CSII based on individual clinical needs, including glycemic variability and hypoglycemia risk [22]. Baseline characteristics of patients with CGM and without continuous glucose monitoring (non-CGM) groups are presented in Supplementary Table S2. There were no significant differences between the two groups in age, sex, BMI, or duration of diabetes. HbA1c was slightly higher in the CGM group than in the non-CGM group. Consistent with the SMBG findings, CGM-derived data showed that the short duration group had higher TIR_CGM_, and lower TAR_CGM_ and MBG_CGM_ compared with the medium and long duration groups after adjusting for the same confounders (Supplementary Table S3).

### 3.5. Factors Affecting Improvement in β-Cell Function Indices After Within 1-Week IIT

Factors influencing improvement in β-cell function indices in these patients were also analyzed. The ISSI-2, which provides a near physiological and comprehensive assessment of β-cell function by taking the interaction between insulin sensitivity and insulin secretion into account, was selected as the primary indicator of β-cell function index improvement for further analysis. Multivariable linear regression analyses were performed to explore factors associated with changes in β-cell function-related parameters following intensive insulin therapy (IIT). The change in ISSI-2 from baseline (Δ ISSI-2) was used as the primary dependent variable, while age, duration of diabetes, BMI, HbA1c, duration of IIT, TIR, and MAGE were included as independent variables. As a sensitivity analysis, the percentage change in ISSI-2 was used as an alternative dependent variable in the same multivariable linear regression model. TIR and MAGE were derived from either seven-point capillary blood glucose profiles (in the entire study population) or CGM data (in the CGM subgroup). The results showed that in the entire study population, age (standardized β = −0.190, unstandardized β = −1.440, 95% CI, −2.029~−0.852, *p* < 0.001), duration of diabetes (standardized β = −0.172, unstandardized β = −0.188, 95% CI, −0.271~−0.104, *p* < 0.001), BMI (standardized β = 0.122, unstandardized β = 3.512, 95% CI, 1.422~5.601, *p* = 0.001), duration of IIT (standardized β = 0.091, unstandardized β = 9.005, 95% CI, 2.334~15.676, *p =* 0.008), and eTIR_SMBG_ (standardized β = 0.108, unstandardized β = 68.895, 95% CI, 16.060~121.731, *p* = 0.011) were significantly associated with Δ ISSI-2. The model explained 16.4% of the variance in Δ ISSI-2 (R^2^ = 0.164, adjusted R^2^ = 0.156; F = 19.872, *p* < 0.001). No substantial multicollinearity was detected among the independent variables, with variance inflation factors (VIFs) ranging from 1.011 to 1.502 and tolerance values ranging from 0.666 to 0.989. In the sensitivity analysis using the percentage change in ISSI-2 as the dependent variable, age (standardized β = −0.205, unstandardized β = −0.017, 95% CI, −0.023~−0.011, *p* < 0.001), duration of diabetes (standardized β = −0.122, unstandardized β = −0.001, 95% CI, −0.002~−0.001, *p* = 0.002), BMI (standardized β = 0.089, unstandardized β = 0.028, 95% CI, 0.006~0.050, *p* = 0.015), HbA1c (standardized β = 0.231, unstandardized β = 0.124, 95% CI, 0.086~0.163, *p* < 0.001), and duration of IIT (standardized β = 0.142, unstandardized β = 0.153, 95% CI, 0.081~0.224, *p* < 0.001) were significantly associated with percentage change in ISSI-2. The model explained 19.8% of the variance in percentage change in ISSI-2 (R^2^ = 0.198, adjusted R^2^ = 0.190; F = 24.975, *p* < 0.001). No substantial multicollinearity was detected among the independent variables, with VIFs ranging from 1.011 to 1.502 and tolerance values ranging from 0.666 to 0.989. Additionally, in the CGM subgroup, age (standardized β = −0.227, unstandardized β = −1.755, 95% CI, −2.492~−1.018, *p* < 0.001), duration of diabetes (standardized β = −0.147, unstandardized β = −0.162, 95% CI, −0.270~−0.055, *p* = 0.003), BMI (standardized β = 0.152, unstandardized β = 4.433, 95% CI, 1.829~7.036, *p* = 0.001), HbA1c (standardized β = 0.106, unstandardized β =5.484, 95% CI, 0.775~10.193, *p* = 0.023), duration of IIT (standardized β = 0.088, unstandardized β = 8.930, 95% CI, 0.445~17.415, *p* = 0.039), and TIR_CGM_ (standardized β = 0.167, unstandardized β = 133.833, 95% CI 49.233~218.432, *p* = 0.002) were all significantly correlated with Δ ISSI-2. The model explained 23.2% of the variance in Δ ISSI-2 (R^2^ = 0.232, adjusted R^2^ = 0.219; F = 18.593, *p* < 0.001). No substantial multicollinearity was detected, with VIFs ranging from 1.011 to 1.619 and tolerance values ranging from 0.618 to 0.989.

**Figure 2:**
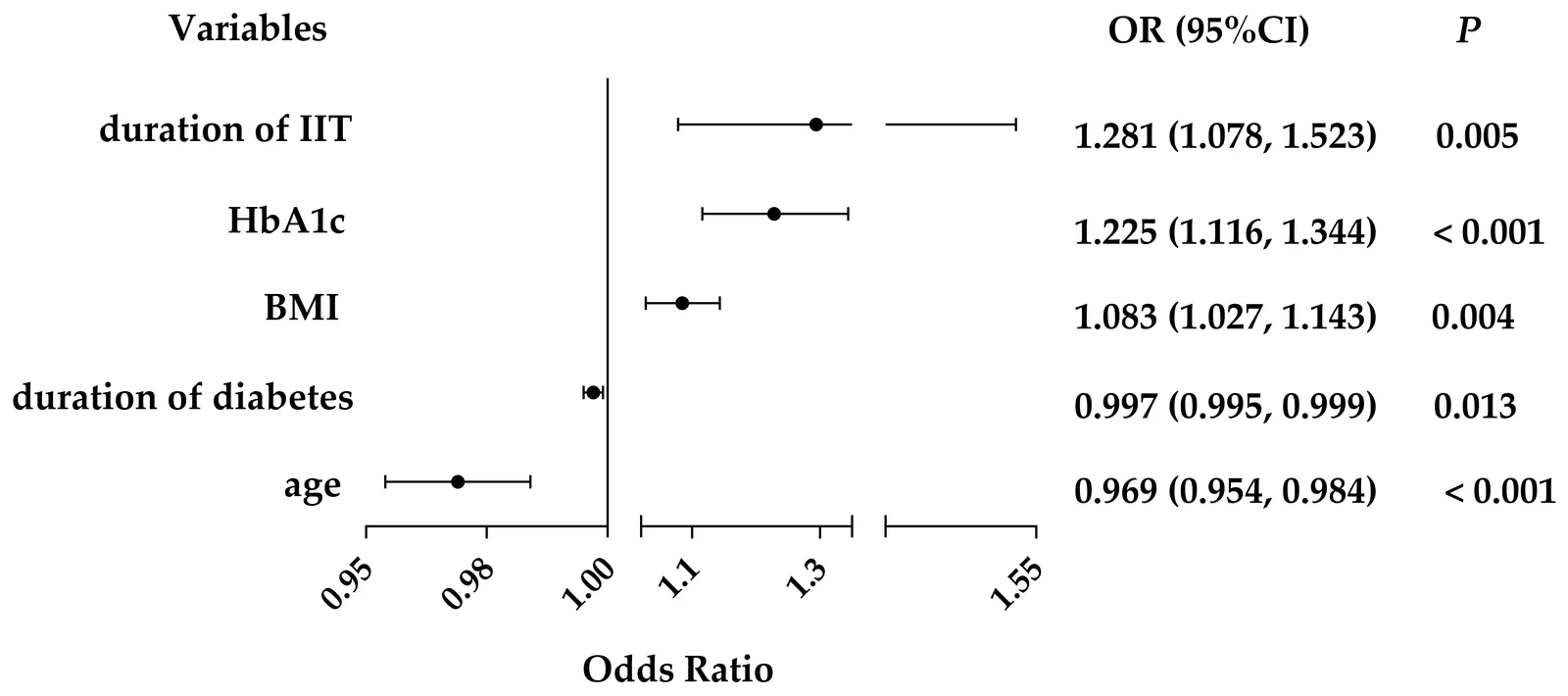
Factors associated with achieving a ≥2-fold increase in ISSI-2 (responders) after intensive insulin therapy in the entire study population were explored using binary logistic regression. IIT, intensive insulin therapy; HbA1c, glycosylated hemoglobin A1c; BMI, body mass index; ISSI-2, insulin secretion sensitivity index-2.

All 726 patients were subsequently categorized into two groups based on their response to IIT. As an exploratory analysis, individuals who experienced a ≥2-fold increase in ISSI-2 after IIT were classified as responders, indicating a favorable β-cell response to treatment (*n* = 218). Conversely, those with a <2-fold increase in ISSI-2 were categorized as non-responders (*n* = 508) [23]. Binary logistic regression analysis was conducted to determine clinical factors associated with β-cell response to IIT, with age, duration of diabetes, BMI, HbA1c, duration of IIT, eTIR_SMBG_, and eMAGE_SMBG_ included as independent variables. The model demonstrated acceptable goodness of fit (Hosmer–Lemeshow test: χ^2^ = 5.581, *p* = 0.694; Nagelkerke R^2^ = 0.191). The model yielded an area under the receiver operating characteristic curve (AUC) of 0.732 (95% CI, 0.692~0.772) for discriminating responders from non-responders. The results demonstrated that age (OR = 0.969, 95% CI, 0.954~0.984, *p* < 0.001), duration of diabetes (OR = 0.997, 95% CI, 0.995~0.999, *p* = 0.013), BMI (OR = 1.083, 95% CI, 1.027~1.143, *p* = 0.004), HbA1c (OR = 1.225, 95% CI, 1.116~1.344, *p* < 0.001), and duration of IIT (OR = 1.281, 95% CI 1.078~1.523, *p* = 0.005) were all significantly associated with the β-cell response to IIT (Figure 2).

### 3.6. Glycemic Control During Follow-Up Among the Three Groups with Different Diabetes Durations

Among the 726 patients included in this study, 213 patients completed a 3–6-month follow-up (Figure 1). Reasons for loss to follow-up included inability to contact the patient, refusal to participate in follow-up, failure to return for scheduled follow-up, relocation or residence outside the study area, loss of contact information, and other reasons. As shown in Table S4, baseline characteristics were compared between patients who completed follow-up and those who did not. All patients showed significant improvements in FPG and HbA1c levels compared with baseline (FPG: 7.18 ± 2.36 mmol/L vs. 9.01 ± 2.74 mmol/L; HbA1c: 7.16 ± 1.62% vs. 9.63 ± 2.01%, respectively; all *p* < 0.001). Similar to the improvement in β-cell function after short-term IIT, FPG and HbA1c levels were more effectively controlled in patients with a shorter duration of diabetes (Table 5).

### 3.7. Incidence of Hypoglycemia Among the Three Groups with Different Diabetes Durations

No significant differences in hypoglycemia were observed among the three groups. The mean numbers of hypoglycemic episodes per patient were 0.32 ± 0.62, 0.33 ± 0.73, and 0.42 ± 0.89, and the proportions of patients experiencing hypoglycemia were 25.00% (52/208), 22.69% (49/216), and 25.17% (76/302) in the short duration group (≤1 year), medium duration group (>1 and <10 years), and long duration group (≥10 years), respectively (all *p* > 0.05).

## 4. Discussion

Previous studies have already demonstrated that short-term IIT could rapidly reduce blood glucose levels and restore damaged β-cell function via elimination of glucotoxicity and other mechanisms. These studies primarily focused on the effects of IIT over durations ranging from 2 weeks to 3 months in patients with T2DM [5,6,8]. Nowadays, short-term IIT is commonly used in patients with both newly diagnosed and established T2DM in real-world clinical practice. However, there is no conclusive evidence regarding the optimal duration of IIT. A previous study found that 1-week IIT with CSII was associated with improvements in FPG and basal β-cell function, as represented by HOMA-β. However, this study mainly focused on the effect of 1-week IIT on the change in inflammatory cytokines and did not evaluate the impact of IIT on β-cell function in a stimulated state. Whether IIT within 1 week could still improve glucose control and enhance both basal and stimulated β-cell function indices in T2DM patients with varying disease durations has not been studied. Consequently, we conducted this retrospective analysis to investigate the effects of IIT within 1 week on glycemic control and β-cell function evaluated by comprehensive indices in T2DM patients, which has not been previously reported. The results from our study suggest that IIT within 1 week significantly alleviates glucose toxicity in T2DM patients with poor glycemic control, especially in those with a disease duration of less than 10 years. Moreover, glycemic variability indices improved most notably in patients whose diabetes duration was less than 1 year.

It is worth addressing that comprehensive indices to evaluate β-cell function in both the fasting and stimulated state with MMTT were adopted in this study. Among these, HOMA-β was used to evaluate basal β-cell function in the fasting state. MMTT is superior to intravenous glucose tolerance (IVGTT) when considering mimicking the physiological process of nutrient absorption [24]. MMTT-derived parameters, including DI_30_, IGI_30_, ISSI-2, Δ Ins_0–120_/Δ Gluc_0–120_ × MI, and AUC_Ins_/AUC_Gluc_ were used to assess stimulated β-cell function in the postprandial state. DI_30_, ISSI-2, and Δ Ins_0–120_/Δ Gluc_0–120_ × MI, which can be derived from the oral glucose tolerance test or MMTT, reflect insulin secretion adjusted for insulin resistance in previous studies [18,25]. Thus, the indicators analyzed in our study offer a comprehensive assessment of β-cell function. Insulin resistance metrics were also evaluated by HOMA-IR and MI. Our results indicate that IIT within 1 week was associated with improvements in several β-cell function-related indices and enhanced insulin-sensitivity-related measures in patients with T2DM, with a more obvious effect on β-cell-function-related indices in those with a shorter duration of diabetes. Importantly, HOMA-β and related indices are influenced by glucose levels and insulin clearance and therefore primarily reflect short-term metabolic and functional changes following IIT, rather than providing definitive evidence of treatment efficacy or restoration of β-cell function.

Our study provides evidence that a shorter duration of IIT within 1 week could still effectively alleviate glucotoxicity and improve both β-cell function and insulin-sensitivity-related measures in T2DM patients with varying disease durations. Moreover, our study also demonstrated that patients with a disease duration within 1 year were expected to benefit more from this treatment regarding glycemic control, glycemic variability, and β-cell function improvement compared with those with a diabetes duration of more than 10 years. Similarly, Retnakaran and his team previously found that a shorter duration of diabetes identifies patients most likely to benefit from 4 to 8 weeks of IIT in patients with a median disease duration of 3 years [26]. It should be noted that the intervention duration of IIT in our study was only 1 week, which is substantially shorter than those reported in previous studies. This therapeutic advantage seems to be more optimal in patients with a shorter duration of diabetes, which may be potentially attributed to their better residual β-cell function and better responsiveness to insulin intervention. Additionally, a positive feedback loop may exist between improved β-cell function and glycemic control measures and the stabilization in T2DM following early short-term IIT. These findings also highlight the importance of timely implementation of IIT in the management of T2DM, both in newly [27] diagnosed and established cases.

Previous research has suggested that younger age at diagnosis of diabetes, shorter duration of diabetes, higher baseline BMI, and higher TIR favored improvements in β-cell function or glycemic remission in patients with T2DM after IIT [6,26,27,28]. Additionally, glycemic fluctuation parameters, assessed by SMBG or CGM, are correlated with β-cell function in patients with T2DM [29,30,31]. Consistent with these findings, our study identified several factors, including age, duration of diabetes, BMI, and TIR, that were associated with improvements of β-cell function. The explanation for these results is as follows: β-cell function tends to decline with age [32], whereas β-cell mass increases with BMI [33]. Additionally, it has been hypothesized that an early window of opportunity may exist in T2DM during which β-cell dysfunction is more amenable to reversal, and that achieving and maintaining near-normal blood glucose levels may potentially facilitate the recovery of β-cell function. In contrast to a previous study [5], our study revealed that a more favorable response to IIT was correlated with higher baseline HbA1c levels. The discrepancy may be attributed to the fact that most patients with higher HbA1c in our study were previously untreated, had a relatively short duration of diabetes, and responded well to IIT, as evidenced by the greater decreases in FPG, 0.5 h PPG, and 2 h PPG observed in these patients. Given the potential for residual confounding in multivariable regression analyses, these factors should be considered exploratory correlates rather than independent causal determinants of improvements in β-cell function-related parameters, and their associations require validation in future prospective studies.

Obviously, the inclusion of a control group [e. g., participants treated with basal insulin or multiple daily injections (MDI)] is important to allow for appropriate comparative interpretation. As previously reported, the efficacy of CSII on glycemic control and β-cell function has already been compared with that of basal insulin [34] or MDI [5]. These studies demonstrated that improvements in β-cell function, represented by multiple indices, were comparable between CSII and basal insulin or MDI. It should be noted that the intervention periods in these studies ranged from an average of 10.5 days to 5 weeks, and the evaluation of β-cell function was a secondary endpoint, which is exactly one of the reasons why we considered it clinically important to evaluate the effect of CSII within 1 week (a shorter intervention period than those used in the previous randomized controlled trials) on β-cell function in real-world clinical practice.

Several limitations need to be indicated. First, the retrospective design of the study may induce bias. To mitigate this limitation, we adjusted for some confounding factors to enhance the reliability of the results. To some extent, we consider that results and information from real-world clinical practice are of significant clinical importance. Second, the patients in the study were hospitalized. Hospitalized patients often have more severe or complicated conditions, which may not accurately represent the general diabetic population. This could limit the generalizability of the findings. Third, due to the modest sample size of the CGM subgroup in our study, we characterized glycemic variability parameters calculated from seven-point capillary blood glucose profiles for all patients. However, the results from the CGM subgroup supported those from the seven-point capillary blood glucose profiles. Finally, the relatively short follow-up period (3–6 months) limits conclusions regarding longer-term outcomes, while differences in baseline characteristics between follow-up completers and non-completers may have introduced selection bias and potentially affected the interpretation and generalizability of the follow-up findings.

## 5. Conclusions

In conclusion, short-term IIT, even within 1 week, was associated with improved β-cell-function-related indices in patients with T2DM, particularly in those with a shorter duration of diabetes. Age, duration of diabetes, BMI, HbA1c, the duration of IIT, and TIR were factors significantly associated with the response to IIT. For patients with T2DM who exhibit poor glycemic control or evident hypermetabolic symptoms, short-term IIT can be administered during hospitalization to improve glycemic levels and β-cell-function-related measures. Future multicenter randomized controlled trials with prospective designs are necessary to compare the efficacy and safety of 1-week IIT and OADs on glycemic control and β-cell function in patients with T2DM to confirm our findings.

## Figures and Tables

**Table 1:** Clinical characteristics of the patients among groups with different durations of diabetes.

	All	Short Duration Group (≤1 Year)	Medium Duration Group (>1 and <10 Years)	Long Duration Group (≥10 Years)	*p*-Value
N	726	208	216	302	
Male, *n* (%)	421 (57.99)	133 (63.94)	125 (57.87)	163 (53.97)	0.081
Age (yrs)	56.28 ± 13.08	49.50 ± 13.49	55.57 ± 12.89 ^†^	61.45 ± 10.47 ^† ‡^	<0.001
BMI (kg/m^2^)	24.57 ± 3.45	25.36 ± 3.58	24.63 ± 3.43 ^†^	24.00 ± 3.26 ^† ‡^	<0.001
HbA1c (%)	9.63 ± 2.01	10.40 ± 2.09	9.55 ± 2.08 ^†^	9.15 ± 1.73 ^† ‡^	<0.001
IIT duration (days)	5.00 (2.00)	5.00 (1.00)	5.00 (2.00)	5.00 (1.00)	0.058
Prior diabetes medications before admission, *n* (%)					
Metformin	331 (45.59)	41 (19.71)	110 (50.93)	180 (59.60)	<0.001
Sulfonylureas *	138 (19.01)	12 (5.77)	49 (22.69)	77 (25.50)	<0.001
Glinides *	20 (2.75)	3 (1.44)	6 (2.78)	11 (3.64)	0.484
DPP-4 inhibitors	149 (20.52)	11 (5.29)	43 (19.91)	95 (31.46)	<0.001
SGLT2 inhibitors	156 (21.49)	16 (7.69)	44 (20.37)	96 (31.79)	<0.001
AGIs	142 (19.56)	13 (6.25)	49 (22.69)	80 (26.49)	<0.001
TZDs	17 (2.34)	1 (0.48)	6 (2.78)	10 (3.31)	0.116
GLP-1RAs *	13 (1.79)	0 (0)	4 (1.85)	9 (2.98)	0.028

Data are presented as mean ± standard deviation or median (interquartile range) for continuous variables or as a numerical proportion for categorical variables. Abbreviations: BMI, body mass index; HbA1c, glycosylated hemoglobin A1c; IIT, intensive insulin therapy; DPP-4, dipeptidyl peptidase-4; SGLT2 inhibitors, sodium-dependent glucose transporters 2 inhibitors; AGIs, α-glucosidase inhibitors; TZDs, thiazolidinediones; GLP-1RAs, glucagon-like peptide-1 receptor agonists. * Sulfonylureas, glinides, and GLP-1RAs were stopped during IIT; ^†^ *p* < 0.05 vs. short duration group; ^‡^ *p* < 0.05 vs. medium duration group.

**Table 2:** Glycemic control at baseline and after IIT among groups with different durations of d diabetes.

	All	Short Duration Group (≤1 Year)	Medium Duration Group (>1 and <10 Years)	Long Duration Group (≥10 Years)
N	726	208	216	302
FPG				
Baseline (mmol/L)	9.01 ± 2.74	9.47 ± 2.59	9.27 ± 2.91	8.50 ± 2.64 ^† ‡^
After IIT (mmol/L)	7.68 ± 1.79 **	7.12 ± 1.50 **	7.48 ± 1.55 **	8.20 ± 1.99 * ^a b^
Δ FPG (mmol/L)	−1.33 ± 2.95	−2.36 ± 2.53	−1.79 ± 3.01	−0.30 ± 2.85 ^a b^
0.5-h PPG				
Baseline (mmol/L)	13.13 ± 3.12	13.71 ± 3.29	13.16 ± 3.01	12.70 ± 3.03 ^†^
After IIT (mmol/L)	10.99 ± 2.41 **	10.26 ± 2.13 **	10.73 ± 2.26 **	11.70 ± 2.52 ** ^a b^
Δ 0.5 h PPG (mmol/L)	−2.14 ± 3.52	−3.46 ± 3.39	−2.44 ± 3.39	−1.01 ± 3.35 ^a b^
2 -h PPG				
Baseline (mmol/L)	14.98 ± 4.16	15.35 ± 4.51	14.76 ± 4.04	14.88 ± 3.99
After IIT (mmol/L)	12.45 ± 3.66 **	11.39 ± 3.27 **	11.99 ± 3.76 **	13.50 ± 3.56 ** ^a b^
Δ 2 h PPG (mmol/L)	−2.53 ± 4.61	−3.95 ± 4.51	−2.77 ± 4.56	−1.38 ± 4.43 ^a b^

Data are presented as mean ± standard deviation. Abbreviations: FPG, fasting plasma glucose; IIT, intensive insulin therapy; Δ, change from baseline; PPG, postprandial plasma glucose. ^*^ *p* < 0.05 vs. baseline; ** *p* < 0.01 vs. baseline. ^†^ *p* < 0.05 vs. short duration group; ^‡^ *p* < 0.05 vs. medium duration group; ^a^ *p* < 0.05 vs. short duration group after adjusting for age, BMI, HbA1c, use of diabetes medications during IIT, and baseline variables; ^b^ *p* < 0.05 vs. medium duration group after adjusting for age, BMI, HbA1c, use of diabetes medications during IIT, and baseline variables.

**Table 3:** β-cell function and insulin sensitivity before and after IIT among patients with different diabetes durations.

	All	Short Duration Group (≤1 Year)	Medium Duration Group (>1 and <10 Years)	Long Duration Group (≥10 Years)
N	726	208	216	302
β-cell function				
HOMA-β				
Baseline	32.40 (46.06)	26.53 (31.99)	31.51 (36.59)	40.80 (82.86) ^† ‡^
After IIT	37.69 (41.03) *	45.05 (44.41) **	37.52 (40.91) ** ^a^	29.42 (34.51) ** ^a b^
IGI_30_				
Baseline	2.12 (3.51)	2.36 (3.78)	3.11 (4.08)	1.59 (2.85) ^† ‡^
After IIT	3.16 (3.97) **	3.89 (4.72) **	3.89 (4.60) ** ^a^	2.48 (2.89) ** ^a^
DI_30_				
Baseline	188.93 (263.45)	202.99 (270.04)	219.01 (254.77)	147.07 (261.89) ^† ‡^
After IIT	336.85 (397.05) **	392.64 (395.86) **	389.99 (405.61) **	256.85 (343.99) ** ^a b^
ISSI-2				
Baseline	120.81 (90.12)	120.49 (77.35)	122.58 (93.07)	120.75 (95.12)
After IIT	185.92 (122.89) **	218.24 (138.17) **	197.83 (127.17) ** ^a^	160.16 (92.02) ** ^a b^
Δ Ins_0–120_/Δ Gluc_0–120_ × MI				
Baseline	190.20 (269.57)	210.55 (297.64)	204.17 (298.01)	168.11 (228.38) ^†^
After IIT	416.65 (440.61) **	470.03 (577.84) **	436.23 (450.73) **	343.51 (380.75) **
AUC_INS_/AUC_GLU_				
Baseline	1.49 (1.68)	1.38 (1.69)	1.56 (1.67)	1.53 (1.67)
After IIT	1.81 (1.71) **	2.19 (1.89) **	1.97 (1.88) ** ^a^	1.53 (1.39) ^a^
Insulin sensitivity				
HOMA-IR				
Baseline	3.25 (3.42)	3.20 (3.21)	3.20 (3.27)	3.35 (3.69)
After IIT	2.29 (2.18) **	2.43 (1.96) **	2.18 (1.88) **	2.24 (2.39) **
MI				
Baseline	80.07 (70.67)	79.12 (72.52)	80.41 (67.01)	80.14 (71.23)
After IIT	104.77 (82.78) **	98.34 (70.66) **	109.44 (85.94) ** ^a^	105.60 (87.31) ** ^a^

Data are presented as median (interquartile range). Abbreviations: HOMA-β, homeostasis model assessment of β-cell function; IIT, intensive insulin therapy; IGI_30_, insulinogenic index 30 min; DI_30_, disposition index 30 min; ISSI-2, insulin secretion sensitivity index-2; Δ Ins_0–120_/Δ Gluc_0–120_, the incremental concentrations of insulin and glucose during mixed meal tolerance test (MMTT); AUC_Ins_/AUC_Gluc_, area under the curve of insulin and area under the curve of glucose ratio; HOMA-IR, homeostatic model assessment of insulin resistance; MI, Matsuda index. * *p* < 0.05 vs. baseline. ** *p* < 0.01 vs. baseline. ^†^ *p* < 0.05 vs. short duration group; ^‡^ *p* < 0.05 vs. medium duration group; ^a^ *p* < 0.05 vs. short duration group after adjusting for age, BMI, HbA1c, use of diabetes medications during IIT, and baseline variables; ^b^ *p* < 0.05 vs. medium duration group after adjusting for age, BMI, HbA1c, use of diabetes medications during IIT, and baseline variables.

**Table 4:** Glycemic variability indices derived from seven-point capillary blood glucose among groups with different diabetes durations.

	All	Short Duration Group (≤1 Year)	Medium Duration Group (>1 and <10 Years)	Long Duration Group (≥10 Years)
N	726	208	216	302
eTAR_SMBG_ (%)	36.75 ± 15.63	33.03 ± 15.30	36.66 ± 15.03 ^c^	39.38 ± 15.78 ^c^
eTIR_SMBG_ (%)	62.41 ± 15.57	66.23 ± 15.12	62.59 ± 15.15 ^c^	59.64 ± 15.64 ^c^
eTBR_SMBG_, % (median [IQR])	0.00 (0.00)	0.00 (1.00)	0.00 (0.00)	0.00 (2.00)
eSDBG_SMBG_ (mmol/L)	2.75 ± 0.76	2.68 ± 0.81	2.72 ± 0.78	2.82 ± 0.69
eLAGE_SMBG_ (mmol/L)	7.16 ± 2.02	6.98 ± 2.20	7.05 ± 2.08	7.37 ± 1.82
eMAGE_SMBG_ (mmol/L)	4.97 ± 1.49	4.85 ± 1.55	4.94 ± 1.52	5.08 ± 1.44
ePPGE_SMBG_ (mmol/L)	4.82 ± 1.66	4.82 ± 1.65	4.76 ± 1.62	4.88 ± 1.70
eCV_SMBG_ (%)	28.91 ± 6.44	28.73 ± 6.77	28.60 ± 6.63	29.29 ± 6.06
eMBG_SMBG_ (mmol/L)	9.47 ± 1.35	9.23 ± 1.40	9.47 ± 1.34 ^c^	9.63 ± 1.29 ^c^

Data are presented as mean ± standard deviation or median (interquartile range). Abbreviations: SMBG: self-monitoring of blood glucose; eTAR_SMBG_, estimated time above range; eTIR_SMBG_, estimated time in range; eTBR_SMBG_, estimated time below range; IQR: interquartile range; eSDBG_SMBG_, estimated standard deviation of blood glucose; eLAGE_SMBG_, estimated largest amplitude of glycemic excursion; eMAGE_SMBG_, estimated mean amplitude of glycemic excursion; ePPGE_SMBG_, estimated postprandial glucose excursion; eCV_SMBG_, estimated glucose coefficient of variation; eMBG_SMBG_, estimated mean blood glucose. ^c^ *p* < 0.05 vs. short duration group after adjusting for age, BMI, HbA1c, and use of diabetes medications during IIT.

**Table 5:** FPG and HbA1c levels after 3–6 months of follow-up among groups with different diabetes durations.

	All	Short Duration Group (≤1 Year)	Medium Duration Group (>1 and <10 Years)	Long Duration Group (≥10 Years)
N	213	87	48	78
FPG (mmol/L)	7.18 ± 2.36	6.51 ± 2.03	7.23 ± 1.85 ^a^	7.90 ± 2.76 ^a^
HbA1c (%)	7.16 ± 1.62	6.30 ± 1.05	7.12 ± 1.17 ^a^	8.16 ± 1.82 ^a b^

Data are presented as mean ± standard deviation for continuous variables. Abbreviations: FPG, fasting plasma glucose; HbA1c, glycosylated hemoglobin A1c; BMI, body mass index. ^a^ *p* < 0.05 vs. short duration group after adjusting for age, BMI, HbA1c, use of diabetes medications during IIT, and baseline variables; ^b^ *p* < 0.05 vs. medium duration group after adjusting for age, BMI, HbA1c, use of diabetes medications during IIT, and baseline variables.

## Data Availability

The data supporting the findings of this study are available from the corresponding author upon reasonable request.

## Supplementary Materials

The following supporting information can be downloaded at https://www.mdpi.com/article/10.3390/jcm15218297/s1, Table S1: computational formulas for β-cell function and insulin sensitivity indices, Table S2: baseline characteristics of CGM group and non-CGM group, Table S3: glycemic variability parameters of patients in CGM subgroup among patients with different diabetes durations, Table S4: baseline characteristics of follow-up completers and non-completers.
