# Supplementary material for: Effects of Intensive Insulin Therapy Within One Week on Glycemic Control and β-Cell Function in Patients with T2DM
Source: J Clin Med. 2026 Oct 25;15(21):8297. doi: 10.3390/jcm15218297 (PMC13764455; doi:10.3390/jcm15218297)
Supplement: Supplementary file 1 [file jcm-15-08297-s001.zip › jcm-4561430-supplementary.pdf]

Supplementary Table S1. Computational formulas for β-cell function and insulin sensitivity indices.

Indices	Detailed computational formulas
HOMA-β	[20 × FINS (mIU/L)] / [FPG (mmol/L) −3.5]
IGI_{30}	(Ins_{30} − Ins_0) (mIU/L) / ($Gluc_{30}$ −$Gluc_0$) (mmol/L)
DI_{30}	IGI_{30} × Matsuda index
ISSI-2	AUC_{Ins} / AUC_{Gluc} × Matsuda index
Δ Ins_{0-120}/ Δ $Gluc_{0-120}$ × MI	(Ins_{120} −Ins_0) / ($Gluc_{120}$ −$Gluc_0$) × Matsuda index
HOMA-IR	FINS (mU/L) × FPG (mmol/L) / 22.5
Matsuda index	$[10000 / \sqrt{FPG\left(\frac{mmol}{L}\right) \times FINS\left(\frac{mu}{L}\right) \times mean\ glucose\left(\frac{mmol}{L}\right) \times mean\ insulin\ \left(\frac{mu}{L}\right)}$

Abbreviations: HOMA-β, homeostasis model assessment of β-cell function; FINS, fasting insulin; FPG, fasting plasma glucose; IGI30, insulinogenic index 30min; DI30, disposition index 30min; ISSI-2, insulin secretion-sensitivity index-2; AUC_{Ins}/ AUC_{Gluc}, area under the curve of insulin and area under the curve of glucose ratio; MMTT, mixed meal tolerance test; Δ Ins_{0-120}/Δ Glu_{0-120}, the incremental concentrations of insulin and glucose during MMTT; HOMA-IR, homeostatic model assessment of insulin resistance.

Supplementary Table S2. Baseline characteristics of CGM group and non-CGM group.

	All	CGM group	non-CGM group	*p*-Value
N	726	442	284	0.679
Male, n (%)	421 (57.99)	259 (58.60)	162 (57.04)	0.750
Age (yrs)	56.28 ± 13.08	56.08 ± 13.40	56.43 ± 12.66	0.480
BMI (kg/m^2)	24.57 ± 3.45	24.50 ± 3.55	24.70 ± 3.29	0.008
HbA1c (%)	9.63 ± 2.01	9.79 ± 2.00	9.38 ± 2.01	0.619
Diabetes duration(yrs)	7.77 ± 7.60	7.68 ± 7.77	7.92 ± 7.32	0.679

Data were presented as mean ± standard deviation or median (interquartile range) for continuous variables. CGM, continuous glucose monitoring; non-CGM, without continuous glucose monitoring; Abbreviations: BMI, body mass index; HbA1c, glycosylated hemoglobin A1c.

Supplementary Table S3. Glycemic variability parameters of patients in CGM subgroup among patients with different diabetes durations.

	All	Short duration group (≤ 1 year)	Medium duration group (> 1 and < 10 years)	Long duration group (≥ 10 years)
N	442	135	128	179
TAR_{CGM} (%)	22.13 ± 14.04	17.94 ± 11.42	23.09 ± 14.96 ^{c}	24.61 ± 14.51 ^{c}
TIR_{CGM} (%)	77.62 ± 12.93	81.55 ± 11.27	77.03 ± 12.72 ^{c}	75.08 ± 13.58 ^{c}
TBR_{CGM}, % (median [IQR])	0.00 (1.00)	0.00 (1.00)	0.00 (1.00)	0.00 (1.00)
$SDBG_{CGM}$ (mmol/L)	2.46 ± 0.66	2.34 ± 0.69	2.50 ± 0.67	2.52 ± 0.63
$MAGE_{CGM}$ (mmol/L)	4.77 ± 1.41	4.57 ± 1.39	4.85 ± 1.54	4.86 ± 1.33
CV_{CGM} (%)	23.98 ± 5.23	23.51 ± 5.42	23.98 ± 5.68	24.32 ± 4.72

MBG_{CGM} (mmol/L)	8.25 ± 1.08	7.94 ± 1.05	8.25 ± 1.08 [c]	8.48 ± 1.06 [c]

Note: Data were presented as mean ± standard deviation or median (interquartile range). Abbreviations: CGM, continuous glucose monitoring; TAR_{CGM}, time above range; TIR_{CGM}, time in range; TBR_{CGM}, time below range; IQR_{CGM}, interquartile range; $SDBG_{CGM}$, standard deviation of blood glucose; $MAGE_{CGM}$, mean amplitude of glycemic excursion; CV_{CGM}, glucose coefficient of variation; MBG_{CGM}, mean blood glucose. [c] $P < 0.05$ vs short duration group after adjusting for age, BMI, HbA1c, and use of diabetes medications during IIT.

Supplementary Table S4. Baseline characteristics of follow-up completers and non-completers.

	All	Patients completed follow-up	Patients lost to follow-up	*p*-Value
N	726	213	513	
Male, n (%)	421 (57.99)	119 (55.87)	301 (58.67)	0.456
Age (yrs)	56.28 ± 13.08	53.12 ± 12.89	57.53 ± 12.98	<0.001
BMI (kg/m^2)	24.57 ± 3.45	24.63 ± 3.44	24.55 ± 3.45	0.746
HbA1c (%)	9.63 ± 2.01	10.07 ± 2.18	9.43 ± 1.91	<0.001
Diabetes duration(yrs)	7.77 ± 7.60	6.53 ± 7.31	8.29 ± 7.66	0.004

Data were presented as mean ± standard deviation for continuous variables. Abbreviations: BMI, body mass index; HbA1c, glycosylated hemoglobin A1c.
